# Financial logistics models based on systematic approach improving management solutions

**DOI:** 10.12688/f1000research.111252.1

**Published:** 2022-05-25

**Authors:** Sergey Evgenievich Barykin, Irina Vasilievna Kapustina, Sergey Mikhailovich Sergeev, Sara Mehrab Daniali, Lyudmila Anatolievna Kopteva, Galina Nikolaevna Semenova, Igor Petrovich Pryadko, Alexey Mikhaylov, Pavel Baboshkin, Polina Datsyuk, Tomonobu Senjyu

**Affiliations:** 1 Graduate School of Service and Trade, Peter the Great St. Petersburg Polytechnic University, St. Petersburg, 195251, Russian Federation; 2 Graduate School of Industrial Management, Peter the Great St. Petersburg Polytechnic University, St. Petersburg, 195251, Russian Federation; 3 Department of Security of High-Tech Systems, St. Petersburg State University of Aerospace Instrumentation, St. Petersburg, 190000, Russian Federation; 4 Department of Accounting and Taxation, Plekhanov Russian University of Economics. Stremyanniy per, Moscow, 117997, Russian Federation; 5 Department of Social, psychological and legal communications, Moscow State University of Civil Engineering (MGSU) National Research University, Moscow, 129337, Russian Federation; 6 Department of Banking and Financial Markets, Financial University under the Government of the Russian Federation, Moscow, 124167, Russian Federation; 7 Department of Electrical and Electronics Engineering, University of the Ryukyus, Okinawa, 903-0213, Japan

**Keywords:** Financial Inclusion, Logistics Models, Cash Balance, Financial Logistics, Optimal Order Size

## Abstract

**Background:** Some firms with good growth opportunities and additional funds could have difficulties accessing external finance. One possible way to enhance their financial inclusion could be an exciting approach to planning the money reserve collected on a firm’s account.

**Methods:** This article aims to disclose the introduction of financial logistics as the new theoretical field of management science. The authors present, in this paper, the key findings on the development of logistical models of an optimum money reserve calculation taking into account digital transformation and industry 4.0 technologies and optimization methods.

**Results:** The monetary reserve models are analogies of models of storekeeping in supply chains. The specific area of the theoretical research of logistics is shown in this paper, which could be disclosed as the subject of financial logistics as a science. The authors consider the term “Financial Logistics” based on logistics theory and money demand.

**Conclusions:** Authors suggest the methodology of studying the nature of both financial and material flows of resources by comparing the relevant formulas. From the researchers’ points of view, financial logistics could be defined as the theory of managing the cash flows based on the logistical models for calculating a corporation’s cash reserve. The authors find it interesting to expand the conditions for calculating financial flows since the uncertainty of external market conditions always influences actual commercial activity.

## Introduction

Currently, industry 4.0 technologies and optimization methods ( Spanos *et al*. 2014; Verny *et al*. 2020; Baruffaldi and Sternberg 2018; Brenner and Hummel 2017; Kayikci 2018; Tjahjono *et al*. 2017) allows to develop and implement new operating models ( Tamás 2018) in various fields of logistics. The authors take into account the broad treatment of the term Financial Logistics, which could stand for multiple areas of consulting activities, including financial solutions for early-stage business's start-up, growth, and expansion, as well as “ building platforms to assist companies in meeting their benchmark results”. The term financial logistics might be considered based on both theoretical points of view: logistics and demand for money.

Logistics can be considered the science of managing material and related information and financial and service flows in an economic system from their origin to the place of consumption to achieve the system's goals with optimal resource costs.

Forming an optimal order batch model is one of the most critical problems of logistics, which is a crucial element of logistics theory in analyzing existing supply chains and designing optimal logistics systems. At the same time, to plan the balance of funds in the current account of the corporation, we can apply the idea of achieving a balance between the cost of placing an order and the cost of maintaining a stock of goods in the warehouse. The research results on the development of logistics models are presented in this article for calculating the optimal cash reserve. This article aims to disclose the introduction of financial logistics as the new theoretical field of management science.

The authors propose the combined approach concerning some critical issues from the theory of money demand by Bennet T. McCallum and Marvin S. Goodfriend ( McCallum and Goodfriend 1987). The improvement of the classical logistical approach to finances is based on the critical papers by Sprenkle ( Sprenkle 1969) (regarding the disadvantages of transactions demand for money models), Meltzer ( Meltzer 1963) (considering the demand for money in relation to business firms), studying of the Maurice Allais’ priority of the Baumol-Tobin optimal cash balance model in the work ( Baumol and Tobin 1989), Tobin ( Tobin 1956) (the interest-elasticity of the demand for cash at a given volume of transactions), Morris ( Morris 1971) (discussing the transactions demand for cash), Grace ( Grace 1975) (examining the specification of the cost function in Baumol's and on Morris' transactions demand for cash), Karni ( Karni 1973) (investigating possible interrelations among the volume of transactions, the rate of interest and the cost of cash withdrawal), Weitzman ( Weitzman 1968) (commenting stochastic approach to exploring demand for money by firms) and cash management models ( da Costa Moraes, Nagano, and Sobreiro 2015). Also, the authors find interesting the idea that the transactions should spend time depending on the technological innovations in the financial sphere. The researchers agree with Orazio Attanasio, Luigi Guiso, Tullio Jappelli ( Attanasio, Guiso, and Jappelli 2002).

The authors suggest comparing formulas for calculating the optimal amount of cash reserve and various models of the optimal order size. Researchers are attempting to disclose a new systematic approach considering the shared nature of the material and financial flows. So, the methods used for defining the optimal order size in supply chain management could be implemented to manage financial flows.

The researchers suggest the following definition of financial logistics from the theoretical point of view: financial logistics could be defined as the theory of managing cash flows based on the logistical models for calculating the corporation’s cash reserve. This article presents the model for determining the monetary reserve, an analogy to the inventory management model in supply chains. The developed approach considers the theoretical fundamentals for comparing the optimal amount of cash reserve and various inventory management models, which improves both the theory and practice of financial planning.

## Evolution of logistics models for cash inventory management

This research aims to develop a systematic approach to managing financial flows based on the theoretical system previously implemented in supply chain management. The authors have implemented the logistics theory into determining a corporation’s cash reserve. We can develop the used methods based on the analogy of the theoretical approach that has been developed in logistics and supply chain management of optimal order size models. The article proves to extend the framework of the theory and methods that are widely known in supply chains management for a new research field spreading over both the financial management models and the logistics approach.

The authors show the specific area of the theoretical research of logistics which could be disclosed as financial logistics as a science.

Logistics began in the 1950s. Although the logistics processes were carried out earlier in economic activity, they were performed separately, without any logistics management concept in the modern sense. The Optimal or Economical Order Quantity (EOQ) model is the most common model of applied logistics theory. According to Steven Nahmias ( Nahmias 2007), interest in mathematical models for inventory management emerged in the first half of the 20th century. At the same time, in 1915, Ford Whitman Harris outlined a simple model for calculating the optimal order size, and this model was analyzed by R. H. Wilson in 1934 ( Wilson 1934) (according to D. Erlenkotter ( Erlenkotter 1989, 1990)).

Since the 1950s, the idea of inventory management based on demand forecasting for individual groups of goods and raw materials is further spread in the sphere of circulation of financial flows.

An article by William Baumol published in the November 1952 issue of the Quarterly Journal of Economics ( Baumol 1952) is the first work in money management. It should be noted that W. Baumol used the idea of minimizing the total costs of registration and storage of inventory, considering the opportunity costs of storing funds and the costs of attracting financial resources. The basic idea of the Baumol model is that there are opportunity costs of keeping money which is the interest income that can be generated on other assets. However, storing cash reserves allows you to reduce transaction costs. When the interest rate increases, the corporation will seek to reduce the funds due to the opportunity costs of storing money.

We can conclude that the period of development of logistics models for managing the corporation's financial resources begins since the publication of W. Baumol’s article, taking into account the opinion of Baumol and J. Tobin about the priority of this model in work ( Baumol and Tobin 1989). In general, we can talk about another area of application of methods and models of logistics as a science of managing not only materials but also financial flows of the corporation.

A study by Merton H. Miller and Daniel Orr is the subsequent work devoted to calculating the optimal cash balance. This study aimed at developing a model for managing cash reserves in an uncertain environment (published in the August 1966 issue of the Quarterly Journal of Economics ( Miller and Orr 1966)). Another study provided the extension of the model for calculating the cash reserve of M. Miller and D. Orr, which was published in Miller and Orr (1968). The stochastic model assumes the probabilistic nature of the behavior of the corporation's cash flows, which is in contrast to the Baumol-Tobin model.

Bernell K. Stone, an Associate Professor of the master’s degree in Commercial and Industrial Activities and Public Administration at Cornell University, in 1972, proposed an extension of the Miller-Orr model, suggesting the possibility of predicting the net cash flow of a corporation ( Stone 1972). The B. Stone model assumes that the corporation can expect the cash flow with a sufficient degree of certainty, in contrast to the stochastic model for calculating the optimal cash balance of M. Miller and D. Orr.

Improving the logistics models of managing the corporation's financial resources has continued since the 1970s. Considering the possibility of postponing the payment to a later date by obtaining a deferral, an extension of the Baumol model was proposed by Rama Sastry, an Associate Professor at the Indian Institute of Research in Bangalore ( Sastry 1970). The model developed by R. Sastry overcomes the disadvantage of the Baumol model of the absence of the possibility of deferred payment. According to the model, the Sastry objective function includes the costs of financial transactions and the opportunity costs of storing funds, as in the Baumol model, and the interest accrued by the corporation's counterparties on loan provided.

In the process of financial management, the corporation can use the credit line model developed by William A. Ogden and Srinivasan Sundaram and published in the Journal of Financial and Strategic decisions in the spring of 1998 ( Ogden and Sundaram 1998). The interest rate on loans, as a rule, exceeds the return on short-term investments. This means that the cost of servicing the loan exceeds the opportunity cost of the funds received through the sale of securities. Borrowing reduces the number of transactions involving the sale of securities made to replenish the cash reserve. The credit line model allows you to calculate the optimal amount of cash received through the sale of securities and attracted by the credit line for a specific time.

It should be noted that financial resources interacting with material flows are also a controlled link and must obey the general laws of the logistics system. Although there are differences in calculating the optimal values of the cash balance and inventory of material resources, the models have some similarities, shown in Table 1. For example, the formula of W. Baumol corresponds to the Wilson formula used to determine the optimal order size in supply chains.

**Table 1.** Formulas for calculating the optimal amount of cash reserve and various models of the optimal order size.

n/a no.	The formula for calculating the optimal amount of cash balance	Optimal order size models in supply chains management
1.	Baumol $R_{\mathrm{opt}}=\sqrt{\frac{2\mathrm{bP}}{E_{D}}}$ , where b is the costs associated with the transaction for the sale of securities, Rub per transaction; *P* is the total volume of transactions, Rub for the period T; $E_{D}$ is the return on financial investments in securities, %.	Wilson's formula (calculation of the economical batch of the order) $Q=\sqrt{\frac{2{\mathrm{AC}}_{0}}{C_{\mathrm{HR}}}}$ , where A is the need for the ordered product within one year; $C_{0}$ is the cost of completing one order, Rub; $C_{\mathrm{HR}}$ is the cost of storing the order, Rub.
2.	Credit line $R_{\mathrm{opt}}=\frac{E_{\mathrm{KR}}M}{E_{D}+E_{\mathrm{KR}}}$ where ${\left(\frac{2\mathrm{Pb}}{E_{D}}\right)}^{\frac{1}{2}}{\left(\frac{E_{D}+E_{\mathrm{KR}}}{E_{\mathrm{KR}}}\right)}^{\frac{1}{2}}$ , where M is the amount of replenishment of the cash reserve, Rub; $E_{\mathrm{KR}}$ is the interest rate per annum for the loan, %.	The optimal order size when the shortage is acceptable $S_{0}={\left(\frac{2{\mathrm{RC}}_{S}}{{\mathrm{TC}}_{1}}\right)}^{\frac{1}{2}}{\left(\frac{C_{2}}{C_{1}+C_{2}}\right)}^{\frac{1}{2}}$ and $q_{0}={\left(\frac{2{\mathrm{RC}}_{S}}{{\mathrm{TC}}_{1}}\right)}^{\frac{1}{2}}{\left(\frac{C_{1}+C_{2}}{C_{2}}\right)}^{\frac{1}{2}}$ Where $S_{0}$ is the optimal stock level at the beginning of a specific interval; $q_{0}$ is the optimal order size; R is the demand for the ordered product for the period T; $C_{S}$ is the order price, Rub; $C_{1}$ is the cost of storing a unit of production per unit of time, Rub; $C_{2}$ is the penalty for lack of a unit of production, Rub.

The correspondence of the symbols in the formulas for calculating the optimal values of stocks of material and financial resources is shown in Table 2.

**Table 2.** Values in the formulas for calculating the optimal margin.

Name of the compared formulas	Correspondence of the values in the formulas for calculating the optimal value			
	Cash reserve in financial management		Order size in supply chains	
	Symbol	Explanation	Symbol	Explanation
Baumol and Wilson	b	Costs associated with the transaction for the sale of securities, Rub per transaction	$C_{0}$	The cost of completing one order, Rub
	P	The total volume of transactions, Rub for the period T	A	The need for the ordered product within one year
	$E_{D}$	Return on financial investments in securities, %	$C_{\mathrm{HR}}$	The cost of storing the order, Rub
The credit line and the optimal order size if the deficit is acceptable	$C_{\mathrm{opt}}$	The optimal cash reserve, Rub	$S_{0}$	The optimal stock level at the beginning of a specific interval
	M	The amount of replenishment of the cash reserve, Rub	$q_{0}$	Optimal order size
	b	Costs associated with the transaction for the sale of securities, Rub. per transaction	$C_{S}$	Order price
	P	The total volume of transactions, Rub for the period T	R	The demand for the ordered product for the period T
	$E_{D}$	Return on financial investments in securities, %	$C_{1}$	The cost of storing a unit of production per unit of time, Rub
	$E_{\mathrm{KR}}$	Interest rate per annum for the loan, %	$C_{2}$	Penalty for lack of a unit of production, Rub

## Methods

### The uniform cash reserve model

All the models considered (the model of calculating the cash reserve with the possibility of multiple financial investments, the model of the cash reserve taking into account lending and various financial assets, and the model of lending and financial investments with a limit on the number of interest payments) are based on the assumption of instant replenishment of the cash reserve. In some cases, the money is gradually transferred to the current account of the corporation. Therefore, it takes time to replenish the balance of funds in the existing version of the corporation.

The assumption of the model of W. Baumol was used. The balance of funds *R*, attracted to replenish the cash reserve, is reduced until stock is entirely exhausted. Consequently, it is assumed that the amount of cash *R _i_* will decrease evenly in the interval *t* and then the subsequent replenishment of money at the end of the interval *t.* *E _d_* denotes the profitability of the *i-*th financial investment. Then, the total opportunity cost of the corporation from the termination of *N* financial investments will be

$$\sum_{i=1}^{N}\frac{{E_{d}}_{i}R_{i}}{2}$$ (1)

In this case, the equality is valid:

$$\sum_{i=1}^{N}R_{i}=R$$ (2)

Since the total cash reserve is equal to the sum of all potential investments (with the amount of *R _i_* each) that remained unrealized.

Suppose the corporation allocates *R* to investments in shares. In that case, it should determine the claims of *l _i_* (as a percentage of *R*), reflecting the number of funds that can be allocated to a specific financial investment, i.e., for *l _i_* in shares of a unit:

$$R_{i}=l_{i}R$$ (3)

Then, the total opportunity cost (analogous to the cost of storing tangible assets), formulated in the form ( 1), is equal to

$$\sum_{i=1}^{N}\frac{{E_{d}}_{i}R_{i}}{2}=\sum_{i=1}^{N}\frac{{E_{d}}_{i}l_{i}R}{2}$$ (4)

Consider the costs of attracting financial resources. In the model of W. Baumol, it is assumed that the costs associated with the transaction for the sale of securities are *b* rubles for the deal. However, there may be several financial assets, so it is logical to assume that the fixed costs of making transactions with different types of investments are not equal and can be indicated by *b _i_* rubles for the deal. Let us consider the case when the transaction costs contain a constant ( *b _fi_* rubles per transaction) and the variable part ( *b _vi_* ∙ *R* rubles per transaction); that is, the transaction costs are equal to

$$b_{i}=b_{\mathrm{fi}}+b_{\mathrm{vi}}R_{i}$$ (5)

Let us make the following assumptions.

**Figure 1.**
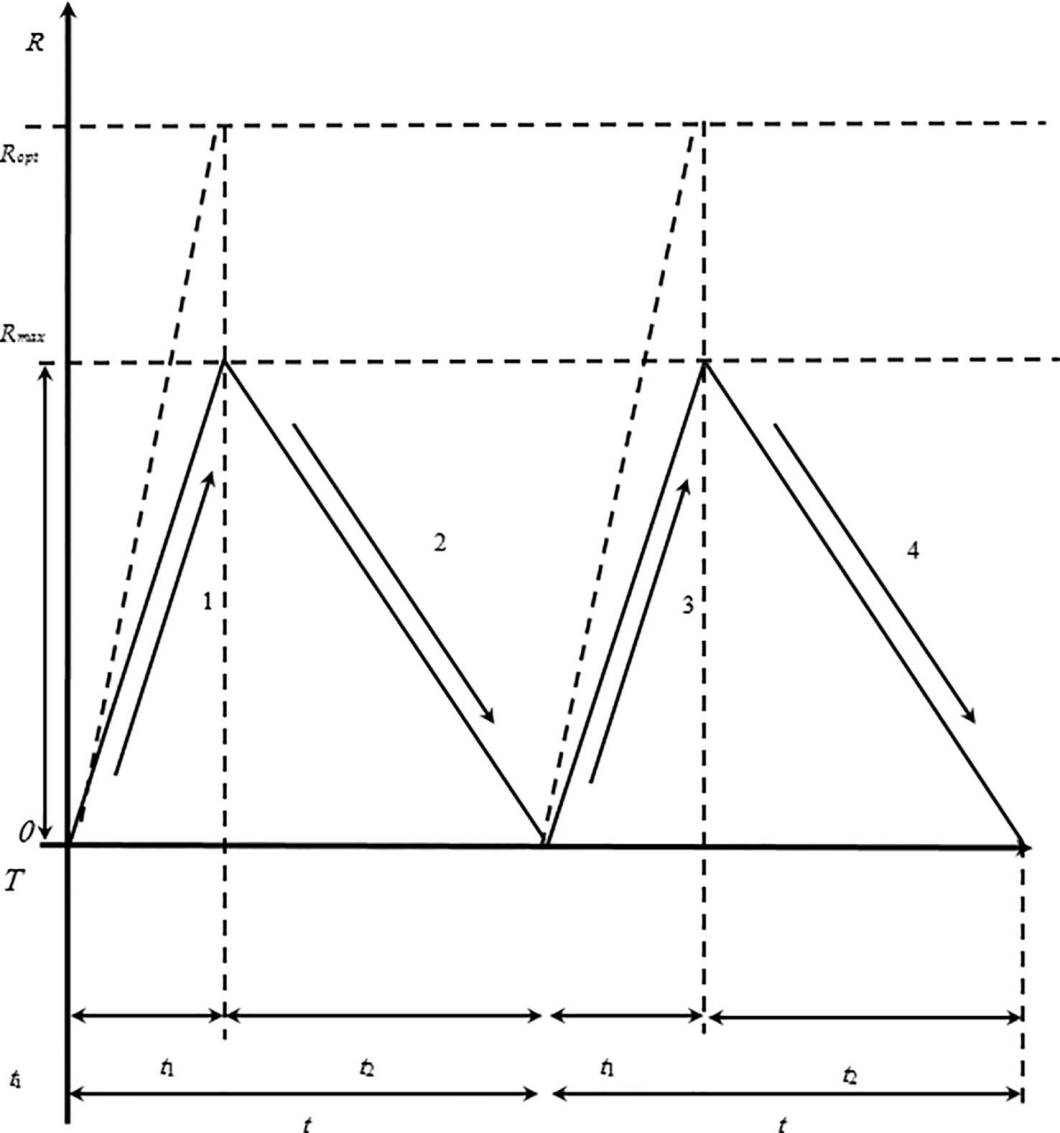
Change in the cash balance according to the proposed cash flow model: 1, 3: proposed cash flow over time at the expense of funds received from the sale of securities; 2, 4: spending of the cash reserve on payments with a total volume of *R.*

1. The corporation uses two types of assets, as in the previous models:
  (a) bank deposits and securities,
  (b) a stock of cash.
2. The corporation’s costs for making transactions with securities and conducting operations for depositing or withdrawing money from a bank deposit do not depend on the transaction volume and include constant and variable parts.
3. The constant intensity of the receipt of funds *u* rubles is known per day during the time interval *t*_1_ ( Figure 1).
4. The corporation that has accumulated a reserve of funds, that is, after the end of the time interval *t*_1_, spends the funds during the interval *t*_2_. At the same time, the intensity of the expenditure of funds is constant throughout the entire time interval *t* ( *t*_1_ + *t*_2_) and is equal to *v* rubles per day.
  Thus, over the period *t _1_*, the reserves increase with an intensity of ( *u* – *v*) rubles per day. After *t*_1_ day, the receipt of funds *R _opt_*, and the balance on the current account starts to decrease with an intensity of *v* rubles per day. The maximum size of the stock is equal to ( *u* – *v*) *t*_1_ rubles for *t*_1_ day.
  Recall that the total costs of the corporation are equal to the sum of the costs of storing and raising funds. Consider the components of the total costs in the interval *t*:
1) the opportunity cost of storing funds will be

$$\frac{R\left(u-v\right)}{2u}\sum_{i=1}^{N}{E_{d}}_{i}l_{i}$$ (6)

2) the cost of raising funds is equal to
$$\sum_{i=1}^{N}\left(b_{\mathrm{fi}t_{1}}+b_{\mathrm{vi}t_{1}}l_{i}R\right)$$ (7)
  where $b_{\mathrm{fi}t_{1}}$ is the fixed costs for raising funds from the sale of type *i* assets during the *t*_1_ interval (transactions with securities), Rub; $b_{\mathrm{vi}t_{1}}$ is the variable part of the cost of raising funds from the sale of kind *i* assets during the *t*_1_ interval, % (converted to unit shares).

Write down the expression for the total costs of the company in the period *T*:

$$F=\frac{P}{R}\sum_{i=1}^{N}b_{\mathrm{fi}t_{1}}+P\sum_{i=1}^{N}b_{\mathrm{vi}t_{1}}l_{i}+\frac{R\left(u-v\right)}{2u}T\sum_{i=1}^{N}{E_{d}}_{i}l_{i}.$$ (8)

Let us take the derivative of *F* concerning *R*, equate it to zero, and get an expression for calculating the optimal size of cash receipts that are uniform over time. *R _opt_* is equal to

$$R_{\mathrm{opt}}={\left(\frac{2P\sum_{i=1}^{N}b_{\mathrm{fi}t_{1}}}{T\sum_{i=1}^{N}{E_{d}}_{i}l_{i}}\right)}^{\frac{1}{2}}{\left(\frac{u}{u-v}\right)}^{\frac{1}{2}}$$ (9)

Having calculated the optimal amount of cash inflow, we write down the expression for calculating the maximum balance of funds on the current account *R _max_*:

$$R_{\max}={\left(\frac{2P\sum_{i=1}^{N}b_{\mathrm{fi}t_{1}}}{T\sum_{i=1}^{N}{E_{d}}_{i}l_{i}}\right)}^{\frac{1}{2}}{\left(\frac{u-v}{u}\right)}^{\frac{1}{2}}$$ (10)

The developed model for calculating the considered cash flow is presented first and analogous to the production order model in supply chains (Economic Production Quantity, EPQ).

### Calculation example

Let us assume that a corporation has the opportunity to purchase securities of various yields (% per year): *E*_*d*1_ = 24 %; *E*_*d*2_ = 16 %; *E*_*d*3_ = 10 %.

Shares (as a percentage of *R*) reflect the amount of funds that can be allocated for a specific financial investment, that is, for *l _i_* in shares of a unit: *l*_1_ = 20 %; *l*_2_ = 35 %; *l*_3_ = 45 %.

At the same time, the fixed costs of making transactions by the corporation are equal, respectively (thousand rubles for each operation): *b*_*f*1_ = 2; *b*_*f*2_ = 3; *b*_*f*3_ = 4.

Variable transaction costs (percentages of the transaction amount) are *b*_*v*1_ = 0,5; *b*_*v*2_ = 0,6; *b*_*v*3_ = 0,65.

The total amount of all payments to the corporation per year is 245,000 thousand Rubles. Moreover, the intensity of receipt of funds is accepted for 1,000 thousand Rubles per day, and the intensity of the expenditure of funds is 671.2 thousand Rubles per day. The funds are spent as they are credited to the corporation's current account.

At the same time, the fixed costs of raising funds from the sale of type *i* assets during the *t*_1_ interval (thousand rubles in the *t*_1_ interval) are $b_{f1,t_{1}}$ =2; $b_{f2,t_{1}}$ =3; $b_{f3,t_{1}}$ =4.

The variable costs of raising funds from the sale of type *i* assets during the *t*_1_ interval are (percentages of the transaction amount) $b_{v1,t_{1}}$ = 0,5; $b_{v2,t_{1}}$ = 0,6; $b_{v3,t_{1}}$ = 0,65.

The model allows us to calculate the optimal receipts and the maximum balance of funds on the current account. Substituting the data in the formulas (9) and (10), we get the optimal value of uniform cash receipts of the *R _opt_* 9,488.2 thousand rubles, and the size of the cash reserve *R _max_* is equal to 3,119.4 thousand Rubles during the year ( Barykin 2022).

Microsoft Excel was used for this calculation example for the developed model.

## Results and discussion

Authors suggest discussing considered theoretical propositions as the introduction to the new field of the research. Studying the latest systematic approach as a subject of financial logistics is suggested.

However, the calculated dependences reflect a particular deterministic case and assume linear profitability and interest rates. The authors find it interesting to expand the conditions for calculating financial flows since the uncertainty of external market conditions always influences actual commercial activity. The reasoning will be carried out by analogy with the calculations of reserves with uncertain demand. The basic equations and parameters also correspond to the financial indicators. To solve such a complicated problem and the list of considered indicators, it is necessary to introduce: $\psi \left(z\right)$ - the probability density function of the required stock of funds z . Then, to carry out the calculation, we will formulate the representation of the problem statement in the following form.

It is necessary to search for a solution that satisfies the condition:

$$\min_{S}\left[b\left(z\right)+L\left(z\right)\right],$$ (1)

where, as before, $b\left(z\right)$ reflects the costs per transaction, written in the general form of a function that allows one to consider the possible nonlinearity of such a relationship. We also represent dependencies in the form of functions $E_{D}\left(z\right)$ and $E_{\mathrm{KR}}\left(z\right)$ .

$L\left(z\right)$ means costs due to uncertainty and are calculated for z≥0 as follows:

$$L\left(z\right)=\int_{0}^{z}E_{D}\left(z-q\right)\psi \left(q\right)\mathrm{dq}+\int_{z}^{\infty }E_{\mathrm{KR}}\left(q-z\right)\psi \left(q\right)\mathrm{dq}.$$ (2)

Since it is also necessary to take into account the variant of the deficit, then for z<0 it can be written:

$$L\left(z\right)=\int_{0}^{\infty }E_{\mathrm{KR}}\left(q-z\right)\psi \left(q\right)\mathrm{dq}$$ (3)

The presented mathematical formalisms are convenient for implementation on a computer. Using any of the add-ons for finding the optimal solution included in all packages of applied programs, the researcher can calculate the result in the form of an optimal solution from the condition:

$$\Psi \left(S_{0}\right)=\int_{0}^{S_{0}}\psi \left(q\right)\mathrm{dq}\geq \theta ,$$ (4)

where θ means the lower limit of the probability of fulfilling the conditions for raising funds, $\Psi \left(S\right)$ means an expression for calculating the cumulative distribution function.

Expressions are much clearer in the case of the discrete nature of monetary assets. Then these formalisms are transformed into sums of the following form:

$$L\left(z\right)=\sum_{0}^{z}E_{D}\left(z-q\right)\psi \left(q\right)+\sum_{z}^{\infty }E_{\mathrm{KR}}\left(q-z\right)\psi \left(q\right).$$

Since the calculation formulas given in Table 1 were built from the condition of linearity of dependencies both $b\left(z\right)$ and $E_{D}\left(z\right)$ as well as $E_{\mathrm{KR}}\left(z\right)$ , applying this assumption, it is possible to solve the resulting equations by differentiating the expression $\left[b\cdot z+L\left(z\right)\right]$ and equating to zero:

$$b+\frac{\mathrm{dL}\left(z\right)}{\mathrm{dz}}=0$$

This equality gives a simple result for linear dependencies: $\Psi \left(S_{0}\right)=\frac{E_{\mathrm{KR}}-b}{E_{\mathrm{KR}}+E_{D}}$ .

The abovementioned expression can be used for approximate estimates, but to calculate economically significant indicators, it is necessary to use an algorithm based on formulas (1) - (4) of this research.

The researchers also suggest discussing the use of the proposed approach from the point of view of financial inclusion as providing greater access to financial services. A financial inclusion policy focuses on both the World Bank and the United Nations Development Programme (UNDP). The World Bank report on Financial Inclusion ( World Bank 2013) describes when some firms have difficulties accessing financial resources due to principal-agent problems or transaction costs. Authors suppose that those firms could implement the proposed financial logistics approach to enhance their financial inclusion to get additional funds to finance working capital and fixed asset investments.

Exploring methods and models of financial logistics in reliance on different conditions could be a topic for future research. The new industry 4.0 technologies (e.g., cyber physics systems, big data, IoT, etc.) allow extending the field of financial logistics implementation based on the digital transformation of logistics ( S. Y. Barykin, Bochkarev, *et al*. 2021; Shmatko *et al*. 2021) and digital twins ( S. Y. Barykin, Kapustina, *et al*. 2021). Authors also argue that studying the developed approach is based on sustainability ( Pouri and Hilty 2018; S. E. Barykin *et al*. 2021; Bevilacqua *et al*. 2020), and the digital transformation concept ( Mikhaylov 2021) is a topic for future research.

## Conclusion

This article presents a model developed by the authors for calculating the cash balance, allowing firms to determine the reserve of funds, enhancing their management solution. The development of financial flow management models based on the logistics methodology, including building analogies with models for determining the optimal order size, will improve planning the balance of funds and increase the efficiency of allocating funds.

The logistics approach is based on the search for a compromise between the fixed costs of transactions (for example, the sale of securities) and the costs of maintaining the cash balance and raising borrowed funds. Various models of cash balance management developed based on the logistics methodology are considered: the model of W. Baumol, the model of debt accumulation, the credit line, and the Miller-Orr model. It can be concluded that although there exist different models for managing the cash balance, there are currently no improvements to the models that allow for the other conditions of the corporation's financial flow planning. The researchers suggest the following definition of financial logistics from the theoretical point of view: financial logistics could be defined as the theory of managing the cash flows based on the logistical models for calculating the firm’s cash reserve.

Authors considered a systematic approach comprising both the theory of financial management and models of optimal order size in supply chain management. The firms can apply the idea of striking a balance between expenses for registration of the order and charges on the maintenance of a stock of the goods in a warehouse. The authors find it interesting to expand the conditions for calculating financial flows and consider the developed approach from the point of view of sustainability concept based on the multidisciplinary approach as a topic for future research, taking into account the uncertainty of external market conditions.

## Data availability

### Underlying data

Figshare: Financial logistics model calculations.xls. https://doi.org/10.6084/m9.figshare.19640694.v1 ( Barykin 2022).

This project contains the following underlying data:

- Data file 1. Financial logistics model calculations.xls (example data with the relevant calculation of the developed model and visualization in MS Excel sheet)

Data are available under the terms of the Creative Commons Attribution 4.0 International license (CC-BY 4.0).
