# Supplementary material for: Financial logistics models based on systematic approach improving management solutions
Source: F1000Res. 2022 May 25;11:570. [Version 1] doi: 10.12688/f1000research.111252.1 (PMC9184927; doi:10.12688/f1000research.111252.1)
Supplement: Peer review history

# Peer review: Reviewer response for version 1

**DOI:** 10.5256/f1000research.122952.r138963

**Published:** 2022-06-24

**Authors:** Sher Zaman Khan

**Affiliations:** 1 Institute of Business Administration, Gomal University, Dera Ismail Khan, Pakistan

Thank you very much for providing me the opportunity to review this article which is submitted by the authors for possible indexing. Actually, the article is good enough. The title of the article is interesting and the overall contents and methodology of the article are rich in multiple aspects, however, I have some minor observations which need to be incorporated by the authors in a revised version of this article. For instance, 

1. The theory of logistics is a broader concept, I want to get it clearer if the authors try to extend the framework of the theory and logistics methods to the sphere of managing financial flows? please clarify.
2. Although the proposed approach is much more interesting than the latest one, however, it needs more clarification in the main text, either, the proposed approach could be implemented in deterministic and stochastic situations?
3. A bit more clarification is needed on the work of William Baumol, published in 1952 entitled “The Transactions Demand for Cash: An Inventory Theoretic Approach.” Is this the first work in “Financial Logistics”?
4. Do the authors assume that the formula of W. Baumol could be considered as an analogy to the Wilson optimal order size formula?
5. I think the authors can link the credit line model to the optimal order size when the shortage is acceptable.
6. Some more explanation is needed in the text on how the optimal order size model in supply chains could help develop the theoretical vision of the cash reserve in financial management?
7. If the authors can add some information on the uniform cash reserve model and how can it be extended to the theory of financial logistics?
8. Authors are supposed to develop the introduction to the new field of research. What could the authors suggest as topics for future research?

In the end, I conclude the entire quality and context of the article are rich enough and can be recommended to be accepted for indexing if the above-mentioned minor improvements are made positively. Regards

Is the work clearly and accurately presented and does it cite the current literature?

Yes

If applicable, is the statistical analysis and its interpretation appropriate?

Yes

Are all the source data underlying the results available to ensure full reproducibility?

Partly

Is the study design appropriate and is the work technically sound?

Yes

Are the conclusions drawn adequately supported by the results?

Yes

Are sufficient details of methods and analysis provided to allow replication by others?

Yes

Reviewer Expertise:

Digital Entrepreneurship, sustainable development goals, financial inclusion, CSR, Strategic Management, Intellectual capital, Entrepreneurial finance, business model innovation, sustainable competitive advantage ect.

I confirm that I have read this submission and believe that I have an appropriate level of expertise to confirm that it is of an acceptable scientific standard.

# Peer review: Reviewer response for version 1

**DOI:** 10.5256/f1000research.122952.r138965

**Published:** 2022-06-09

**Authors:** Inna Evgenievna Barykina

**Affiliations:** 1 Vyborg branch, Herzen State Pedagogical University of Russia, St Petersburg, Russian Federation

After reviewing the research paper I paid attention to the scientific novelty of considering the financial logistics from theoretical points of view: logistics and demand for money. Authors define financial logistics as a new scientific field that could be described as the theory of managing the cash flows based on the logistical models. So, I can make a conclusion that the authors attempt to develop the approach for implementing the logistics theory for calculating the corporation’s cash reserve. Authors explore the model for determining the monetary reserve as an analogy to the inventory management model in supply chains. The findings of the authors are very interesting from a theoretical and practical point of view, but I’d suggest making some improvements. The authors should incorporate the following suggestions and revise the manuscript.

 Reviewer’s Comments:

1. The abstract needs revision and restructuring. The abstract should be revised by adding the aim and future scope of the proposed work.
2. The term “Financial logistics” is used in the title as well as in the abstract. Does this definition stem from logistics or from the theory of financial management? It should be explained in the introduction.
3. The uncertainty of market conditions is a wide concept that is required to explain in this paper’s context.
4. It is advised to explain the nature of the financial logistics in the introduction section to understand the research problem.
5. Paper novelty should be added at the end of the Introduction.
6. It is advised to explain the logical connection between financial logistics and the digital twin concept in the discussion section.

Is the work clearly and accurately presented and does it cite the current literature?

Yes

If applicable, is the statistical analysis and its interpretation appropriate?

Yes

Are all the source data underlying the results available to ensure full reproducibility?

Yes

Is the study design appropriate and is the work technically sound?

Yes

Are the conclusions drawn adequately supported by the results?

Yes

Are sufficient details of methods and analysis provided to allow replication by others?

Yes

Reviewer Expertise:

Social Sciences

I confirm that I have read this submission and believe that I have an appropriate level of expertise to confirm that it is of an acceptable scientific standard.

# Peer review: Reviewer response for version 1

**DOI:** 10.5256/f1000research.122952.r138964

**Published:** 2022-06-07

**Authors:** Mohammad Maruf Hasan

**Affiliations:** 1 School of International studies, Sichuan University, Chengdu, China

Thank you so much for giving me the opportunity to read your article. It’s a good informative study, however, some points need to be clarified and suggestions incorporated.

1. Do the authors mean that the term financial logistics could refer to the wide range of solutions based on the logistics theory and demand for money? This point needs a bit more elaboration in the text.
2. The author needs to elaborate on how the logistics approach could be implemented in financial solutions?
3. More explanation is needed on what could be considered a comprehensive treatment of the term “Financial Logistics”?
4. Could an optimal order batch model be considered as a basis for a theoretical approach for some financial models?
5. Can the authors prove that logistics models could be the fundamental base for calculating the optimal cash reserve of the company?
6. The logistic model and theory of money of demand seem to be ambiguous in the text. Does the proposed combined approach include the logistics models and the theory of money demand?
7. Can you please suggest your definition of financial logistics?
8. My final question is, Could specialists in financial analytic managing financial flows implement the methods used for defining the optimal order size in supply chain management?

Is the work clearly and accurately presented and does it cite the current literature?

Yes

If applicable, is the statistical analysis and its interpretation appropriate?

Partly

Are all the source data underlying the results available to ensure full reproducibility?

Yes

Is the study design appropriate and is the work technically sound?

Yes

Are the conclusions drawn adequately supported by the results?

Yes

Are sufficient details of methods and analysis provided to allow replication by others?

Yes

Reviewer Expertise:

NA

I confirm that I have read this submission and believe that I have an appropriate level of expertise to confirm that it is of an acceptable scientific standard.
